# Type 4 Ileal Atresia and Anorectal Malformation in a Neonate: A Rare Association

**DOI:** 10.7759/cureus.24295

**Published:** 2022-04-19

**Authors:** Munema Khan, Jawad Abbasi, Noor Ul Sabah, Mudassar Gondal, Jawad Basit

**Affiliations:** 1 General Surgery, District Headquarter Hospital, Rawalpindi, PAK; 2 Pediatric Surgery, Holy Family Hospital, Rawalpindi, PAK; 3 Medicine, Holy Family Hospital, Rawalpindi, PAK

**Keywords:** pediatric surgery, neonatal intestinal obstruction, pelvic kidney, imperforate anus, jejunoileal atresia

## Abstract

Anorectal malformations and jejunoileal atresias are common causes of intestinal obstruction in neonates. Both have their own set of associated anomalies but it is extremely rare for the two to co-occur in the same patient. In this case report, we detail and describe this unusual incidence in a three-day-old neonate who was provisionally diagnosed with a case of simple imperforate anus. Per-op findings showed a type 4 Ileal atresia and an ileostomy was then created. Our experience stresses the importance of timely antenatal diagnosis and the presence of a high index of suspicion when encountering such patients. Both factors are key and crucial in determining the outcome and post-op course of the patient.

## Introduction

Ileal atresia is a widely prevalent cause of neonatal intestinal obstruction, observed in 95% of cases [1]. Most of these patients present within the first 24 hours of life and the course henceforth is relatively straightforward. Cases of associated cystic fibrosis and cardiac anomalies are well known [2]. However, anorectal malformations (ARMs) in conjunction are exceedingly rare and barely a few cases have been reported in English literature [3]. Both anomalies stem from unrelated embryological development and harbor their own set of clinical peculiarities [4]. A causal linkage is yet unelicited and obscure. The combination, thus, presents not only an etiological enigma but also a unique diagnostic challenge entailing a protracted therapeutic pathway. In a third-world country where antenatal care access and evaluation are restricted as well as limited, the challenges are further amplified. In this case report, a three-day-old neonate is documented who presented to us with a diagnosis of imperforate anus. An uncommon type 4 ileal atresia and pelvic kidney were discovered perioperatively. Our experience serves as a primer for future surgeons who ought to be well aware and well prepared when presented with such antenatally neglected and unprepared cases.

## Case presentation

**Figure 1:**
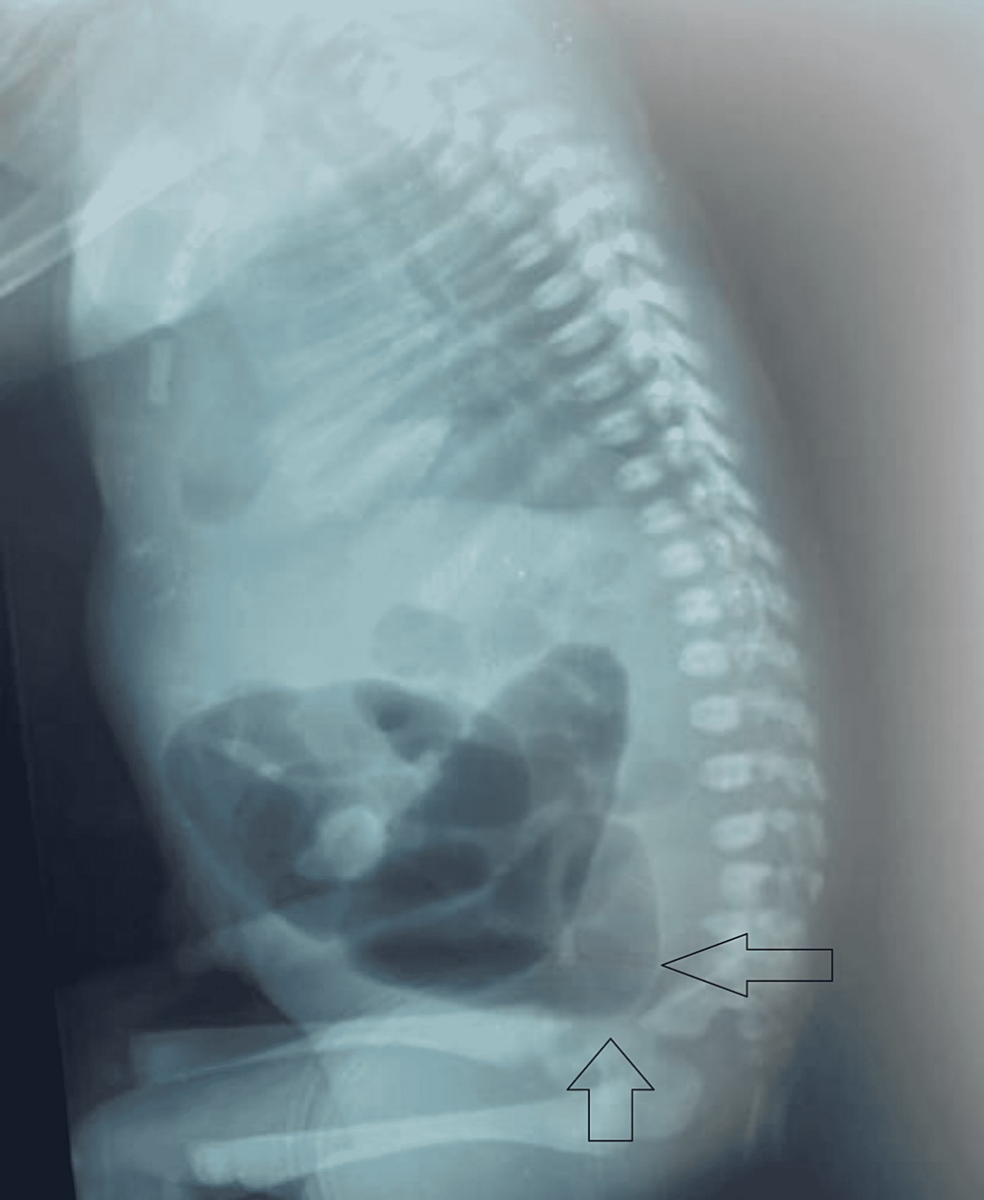
Abdominal X-ray; lateral decubitus view shows dilated gut loops with absence of air at the site of rectum near pelvis.

A two-day-old full-term male neonate presented to the emergency department with complaints of abdominal distension, repeated episodes of bilious emesis, and absent passage of meconium since birth. He was delivered at a primary healthcare setup via spontaneous vaginal delivery and subsequently referred on the diagnosis of an ARM. At the time of admission, the neonate's heart rate was 142/min. His respiratory rate was 32/min and he was afebrile. Abdominal examination revealed a tense, distended, and tender abdomen. Bowel sounds were absent. On perineal examination, the anal opening was notably absent and a bucket handle deformity was present. Chest auscultation reported bilateral crepitations. Routine blood work and radiography were done. The complete blood parameters were within the normal range. The serum potassium was elevated at 5.9 mEq/L and a de-potash was done with nebulized salbutamol. Renal function tests were normal and liver function tests showed neonatal hyperbilirubinemia (bilirubin level 7.5 mg/dL). X-ray of the erect abdomen demonstrated multiple air-fluid levels within a distended gut (Figure 1). Ultrasonography of the abdomen reported obscuration of the mid-abdomen by bowel gases and minimal-to-mild pelvic ascites. 

Preoperative optimization was carried out with good hydration, normalization of lab parameters, and decompression of the gut by the placement of a nasogastric tube. Oxygen inhalation was also instituted as the oxygen saturation was in the 80-86% range due to probable aspiration. After evaluation and approval from the anesthetist on call, the neonate was shifted for exploratory laparotomy with a plan of sigmoid colostomy at present with the definitive procedure to be carried out on an elective list after improvement of the physiological reserve of the neonate. 

**Figure 2:**
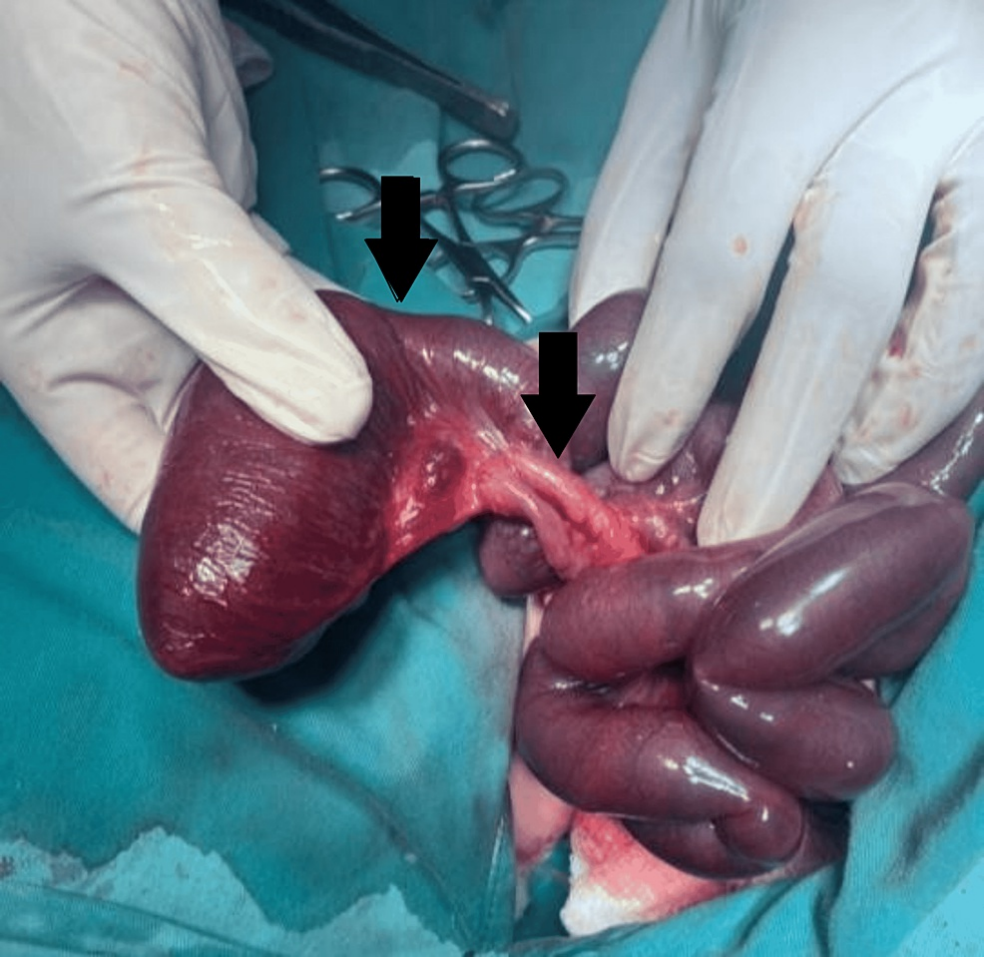
Proximal ileal atresia; the figure demonstrates the presence of proximal ileal atresia, and thinning and shortening of mesentery is seen throughout different segments of the small intestine. The overall gut is dusky and did not improve on oxygenation and warm packing indicating a poor prognosis.

A transverse supra-umbilical laparotomy incision was made. The gut was examined to determine the site of the sigmoid colostomy. Type 4 Ileal atresia was discovered with multiple atretic and dilated segments in the ileum (Figure 2).

**Figure 3:**
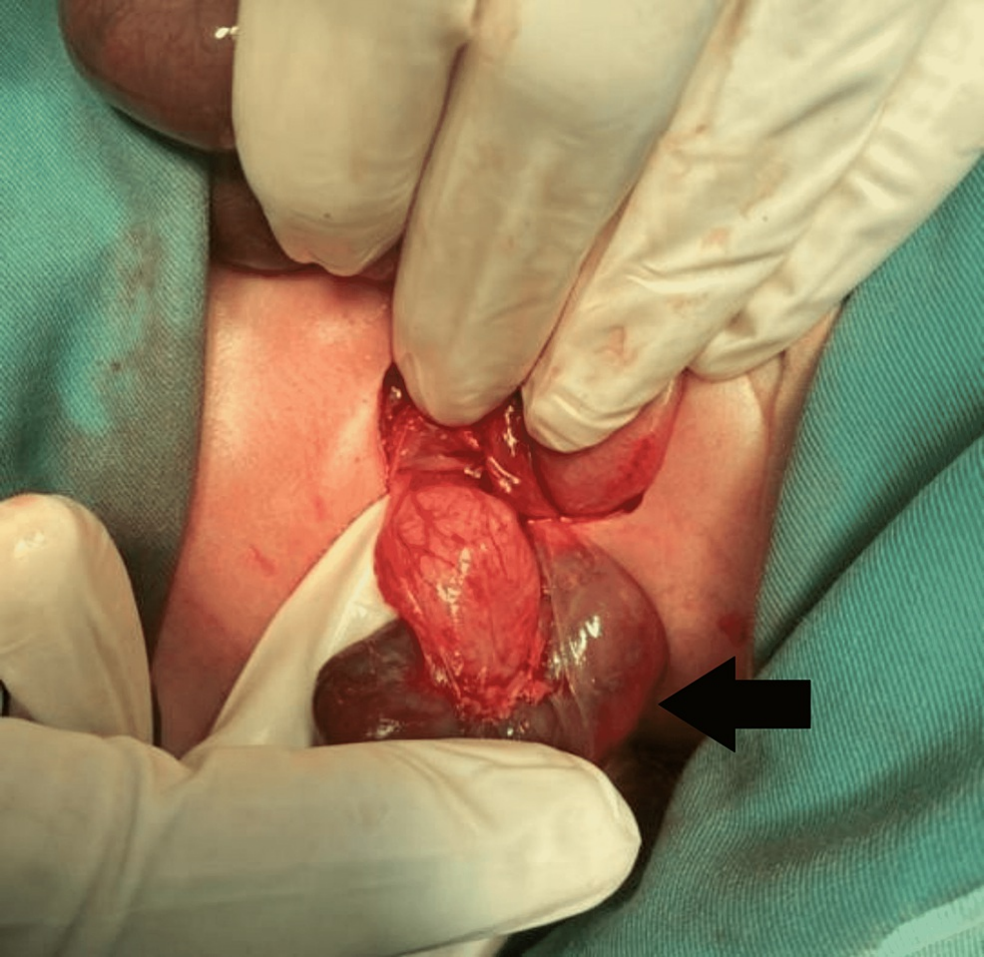
Pelvic kidney, palpated near left iliac fossa and delivered. The figure demonstrates renal pelvis along with abnormally dilated left ureter.

In addition to this incidental finding, a solid organ was appreciated to be present at the level of the left iliac fossa. The presence of renal hilum and ureter was noted and a left pelvic kidney was confirmed (Figure 3). The contralateral kidney was palpated in a normal position. 

As already diagnosed, ARM was visible. The sigmoid colon was found to be normal. An ileostomy and mucus fistula were then created. On examination, thinning of the mesentery was noticed. The absence of mesentery was also appreciated along various lengths of the gut (Figure 2). The perfusion of the gut was normal on exposure but dusky discoloration of the gut was observed that immediately followed any form of light handling. Warm, saline packs with oxygen inhalation were repeatedly placed to counter the occurrence and the gut would transiently respond with recurrent discoloration on handling. No perforation, serosal tear, or any other anomaly such as volvulus associated with malrotation was found.

Bilious emesis as a clinical presentation is inconsistent with the diagnosis of a distal bowel obstruction. Ideally, this should instigate an immediate search for concurrent proximal pathology. However, in our case, given the 1) physiological state the neonate presented in due to late presentation and 2) due to limited availability of investigative facilities in our emergency setup, it was deemed best to go ahead with an operative diagnosis and not delay decompression. Proximal pathology such as volvulus or malrotation, however, was, respectively, ruled out on exploration.

The neonate had an uneventful reversal from anesthesia and once cleared from the recovery room was shifted to the high dependency unit for observation and further management. The child was vitally stable and all postoperative laboratory parameters and measures were satisfactory. Antibiotics, fluid, and IV analgesics were administered. Stoma was functional and oral feed was started on the second postoperative day. The bilateral chest crepts failed to resolve and fluid overload was suspected and judiciously managed. The patient was shifted to the neonatal intensive care unit and entered a critical state. On the second postoperative day, the stomal margins exhibited dusky changes and the patient was oxygen-dependent. On the third postoperative day, unfortunately, the neonate expired. Lack of physiological reserve and ability to withstand the trauma of surgical intervention along with the gut-related pathology were theorized as causal factors for the demise of the patient.

## Discussion

Jejunoileal atresia is a major cause of intestinal obstruction in neonates, reported in one in 5000 to one in 14,000 live births [5]. Most cases present within the first 24 hours of birth; however, depending upon the location of the lesion, delayed presentation is also noted. ARMs, spanning a varied spectrum of anomalies surmised to an imperforate anus, are a similarly prevalent defect sharing a comparable incidence of one in 5000 live births. Unlike the former, these host a gender predilection to a male cohort [6]. The co-occurrence of both anomalies in the same patient is extremely rare and probes consequent etiological inquiry. ARM confers a masking effect neglecting the co-existing atresia which must be sought out underscoring/highlighting the importance of having a high index of clinical suspicion. Though alimentary atresias harbor a good outcome, adverse outcomes continue to be a cumbersome challenge in compromised setups.

While the incidences of both pathologies mirror each other and the common general clinical presentation akin to features of intestinal obstruction are convergent, markedly contrasting features are characteristic of each disorder, respectively. Currently, the co-existence is not theorized to be interlinked, and independent embryological events involving both mid- and hindgut are implicated.

Bilious emesis is indicative of a proximal bowel pathology and it is imperative that this should be sought out and urgently characterized during preoperative workup. CT scan abdomen and pelvis, upper gastrointestinal contrast study, and ultrasound are of use for the purpose and will aid in the accurate and timely diagnosis of a duodenal or small bowel obstruction as well as volvulus or malrotation. The presence of proximal disorders must be borne in mind and investigated appropriately guided by the physiological status of the patient. The latter permits operative exploration if unsatisfactory.

Sixty percent of newborns with ARMs have an associated anomaly while only 10% of ileal atresias bear an extra-abdominal association [5]. The former, thus, warrants a thorough physical evaluation with multiple imaging modalities mandatory. These encompass but are not limited to the VACTERL set of pathologies. Jejunoileal atresia in contrast is linked with cardiac abnormalities, gastroschisis, cystic fibrosis, and malrotation [2,4,5,7]. Notably, these additional anomalies are observed predominantly with jejunal and then ileal cases. In our case, we were unable to rule out the associated anomalies due to limitations imposed both by the postoperative status of the patient as well as the adjunctive investigative resources (echocardiography, ultrasonography [USG], radiology, etc) being located at a considerable distance within the premises and not portably available. 

The association between the two anomalies in our study is extremely unusual, and when reported, the frequency of colonic atresias outnumbers those of the small bowel [3,8,9]. We have found only three case reports in the literature review of a dual diagnosis documenting a total of less than 10 such cases [10-12]. In a 10-year study conducted from 2006 to 2015, out of 950 cases of ARM only two were associated with jejunoileal atresia. One of the two cases expired postoperatively [13]. One study reports the co-occurrence with a type 3A atresia. The authors document a resulting volvulus on re-exploration due to failure to identify the atresia earlier on and proceeding with only ARM repair. Five out of six cases in one study underwent a second laparotomy due to a similar failure to identify the lesion in the first instance. This misdiagnosis amplifies the toll of the surgery, increasing the operative morbidity and recovery course. This association, thus elucidated in the present study, should illuminate the outlook of a pediatric surgeon when encountering such cases and stresses the importance of prenatal imaging and clinical correlation. The patient in our study presented from a limited prenatal setup with inadequate antenatal check-ups and diagnostics imaging.

ARMs have an established genetic etiology with formative deviations in cloacal development [6]. Given that the cloaca is the common canal for urological, rectal, and genital systems, all three systems can be affected depending on the severity of the disease. Ileal atresia, commonly jejunoileal atresia, on the other hand, has been extensively elaborated and attributed to in utero vascular disruption with the midgut mesenteric branches being the incriminating vessels. The impaired perfusion results in ischemic necrosis and resultant resorption of the affected segment of the bowel leaving an associated mesenteric defect in tow. An environment-based causal relationship has also been reported between mothers exposed to vasoconstrictive drugs and smoking in the first trimester of pregnancy. Employing the same etiological mechanism, thromboembolic events in utero can also initiate a similar causal cascade [14]. The mother of the child in our study, however, had none of these risk factors present, with the only unusual finding being the parents of the neonate having a consanguineous marriage. 

Ileal atresia is classified into five types, and the one present in our case was type 4 which is constituted of multiple areas of atresia. This is a relatively rare type carrying the worst survival rate of 29% compared to 81% of type 1 and others [15]. Type 4 ileal atresia is linked with short bowel syndrome, central nervous system dysfunction, and severe immunodeficiency in 25% of the cases [16].

Prenatal screening is instrumental and a detection rate of 29-50% has been reported via USG [4,7]. Shah et al. enumerate diagnostic clues that can alert the clinician to suspect a dual anomaly as a) the presence of mucus with no meconium at anoplasty or colostomy, b) microcolon at colostomy, c) inconsistency in clinical and radiological findings, and d) associated polyhydramnios and ascites [17]. Timely diagnosis can enable both parents and doctors to intervene appropriately and greatly impact the associated morbidity and mortality. Aspiration, electrolyte imbalances, and emesis resulting from the early oral feed are all avoidable caveats that can in cases, such as the one present in our study, be the decisive and determinant factor in the ultimate outcome of the patient.

Following preoperative optimization, the surgical treatment undertaken in ileal atresias depends upon the type of the lesion and the length and state of the unaffected bowel. Ideally, a primary resection anastomosis is done with or without a tapering enteroplasty of the proximal bowel [18]. In instances where anatomical condition (size discrepancy, viability) is not permissive, an ileostomy can be constructed with a definitive procedure scheduled later on [4]. In our case, the planned surgery was a sigmoid colostomy with the definitive procedure of the ARM electively guided by the type of lesion. We created an ileostomy given the precarious viability of the bowel coupled with the multiple atretic segments requiring multiple anastomoses. The latter was deemed to fare better after the postoperative build-up of the neonate.

Advances in prenatal diagnosis, pediatric anesthesia, surgical technique, and neonatal intensive care have greatly improved the prognosis of such cases; however, it remains a potentially life-threatening malformation with a mortality rate as high as 11-70% reported in low- and middle-income countries [4,18]. The postoperative course warrants close follow-up, often punctuated with short bowel syndrome and adhesive obstruction. The former and the associated cardiac anomalies are the main harbingers of a fatal outcome, incurring prolonged parenteral nutrition and a high dependency state with indoor hospitalization. A study reported the occurrence of adhesive obstruction in all cases of jejunoileal atresia within one year of correction [7].

In our present study, several unfortunate pitfalls include 1) lack of preoperative characterization of the disease, 2) delayed presentation of the patient with developing peritonitis, 3) aspiration-associated respiratory compromise due to unchecked oral feed, and 4) physiological depletion of the neonate due to consequent emesis, and all these contributed to a fatal outcome. The authors aim for their experience to 1) shed light on the extremely rare association between ARM and type 4 ileal atresia, furthering the understanding and relevant literature on the subject; 2) educate and sensitize other surgeons working in a similar setup who might be presented with a similar child in emergency practice. It is pertinent that other intestinal obstructions/atresias are ruled out whenever a pediatric surgeon is confronted with a child with a straightforward ARM. The few case reports on the topic exhibit an unacceptably high rate of a second laparotomy for the true diagnosis. Clinical suspicion and judicious examination per-op of each case are imperative to avoid the same missteps that many neonates cannot tolerate or physiologically afford.

## Conclusions

Jejunoileal atresias have not been reported to have a significant association with ARM as well as renal disorders. This case report emphasizes the co-existence of all three pathologies in neonates and their possible effect on the prognosis. What needs to be reviewed is that 1) ARM, renal abnormalities, and ileal atresia when occurring together can be grouped into a specific syndrome or should be labeled as different primary pathologies occurring simultaneously. 2) Approaching the disease methodologically will help us rule out its genetic predisposition as well as a source of common embryological insult affecting the small gut as well as hindgut. 3) A sign that can be interpreted from the clinical presentation is the presence of biliary emesis in a neonate with ARM. This should be investigated appropriately and a proximal pathology must be ruled out. 4) The role of prenatal diagnosis using ultrasonography always forms the basis on which further management options should be planned. This emphasizes the need for proper counseling of the caregivers regarding feeding practices and prompt treatment. The timely presentation along with relevant examinations and investigations leading to the possibility of dual diagnosis and preoperative standardized resuscitation will help improve the outcomes of such conditions remarkably. In a third-world developing country like Pakistan, a review of the epidemiology of different gastrointestinal abnormalities in neonates is imperative.
